# Compounds targeting ferroptosis in breast cancer: progress and their therapeutic potential 

**DOI:** 10.3389/fphar.2023.1243286

**Published:** 2023-10-18

**Authors:** Chuchu Xu, Yian Chen, Qinghong Yu, Jiaqing Song, Ying Jin, Xiufei Gao

**Affiliations:** ^1^ The First Clinical Medical College of Zhejiang Chinese Medical University, Hangzhou, Zhejiang, China; ^2^ Department of Breast Surgery, The First Affiliated Hospital of Zhejiang University of Traditional Chinese Medicine, Hangzhou, Zhejiang, China

**Keywords:** breast cancer, ferroptosis, ferroptosis-inducing agents, drugs, natural compounds

## Abstract

In recent years, there has been a significant increase in the incidence of Breast cancer (BC), making it the most common cancer among women and a major threat to women’s health. Consequently, there is an urgent need to discover new and effective strategies for treating BC. Ferroptosis, a novel form of cell death characterized by the accumulation of iron-dependent lipid reactive oxygen species, has emerged as a distinct regulatory pathway separate from necrosis, apoptosis, and autophagy. It is widely recognized as a crucial factor in the development and progression of cancer, offering a promising avenue for BC treatment. While significant progress has been made in understanding the mechanisms of ferroptosis in BC, drug development is still in its early stages. Numerous compounds, including phytochemicals derived from dietary sources and medicinal plants, as well as synthetic drugs (both clinically approved medications and laboratory reagents), have shown the ability to induce ferroptosis in BC cells, effectively inhibiting tumor growth. This comprehensive review aims to examine in detail the compounds that target ferroptosis in BC and elucidate their potential mechanisms of action. Additionally, the challenges associated with the clinical application of ferroptosis-inducing drugs are discussed, offering valuable insights for the development of novel treatment strategies for BC.

## 1 Introduction

According to the latest global cancer statistics, the incidence of Breast cancer (BC) has reached the first place among all cancer types in women, surpassing lung cancer (Sung et al., 2021). The current treatment options for BC include surgery, radiotherapy, chemotherapy, hormonal therapy, and targeted therapy (Gradishar et al., 2022). These therapies have significantly enhanced the survival rates for BC patients. Nevertheless, approximately 10%–15% of patients with early-stage BC still face the risk of local recurrence during long-term follow-up. Tragically, some of these patients succumb to tumor progression or treatment failure (Sopik et al., 2016). Therefore, it is crucial to find more effective treatment strategies for BC. Recent research has highlighted the significant role of ferroptosis in various diseases, such as neurodegenerative diseases (Yao M-Y. et al., 2021), cancer (Zhang C. et al., 2022), cardiovascular diseases (Wu et al., 2021), and chronic kidney disease (Zhou Y. et al., 2022). Ferroptosis, a distinct form of cell death separate from apoptosis, autophagy, and necrosis, is characterized by excessive accumulation of lipid hydroperoxides to lethal levels in iron-dependent pathways (Tang and Kroemer, 2020). Mounting evidence suggests that ferroptosis inhibits tumor cell growth, making it a promising target for anticancer therapy (Chen et al., 2021). Significant progress has been made in recent years regarding ferroptosis research in BC, particularly in understanding its mechanisms. For instance, Xie Y et al. (Xie et al., 2022a), discovered that mammary adipocytes could protect BC cells from ferroptosis via oleic acid in the presence of ACSL3. Additionally, the role of non-coding RNA has been elucidated in BC, which includes circular RNA, microRNA, and lncRNA, in BC development (Mao et al., 2018; Zhang H. et al., 2021; Bazhabayi et al., 2021; Zhang et al., 2023). However, there are currently no drugs specifically designed for ferroptosis in BC. Consequently, exploring drug-induced ferroptosis in BC represents a promising and valuable avenue of research (Sui et al., 2022). This review aims to summarize the compounds capable of inducing ferroptosis in BC, encompassing both natural phytochemicals and chemically synthesized drugs. It is expected to provide valuable insights for future drug development in the field of ferroptosis induction for BC.

## 2 Mechanism of ferroptosis in BC

The regulation of ferroptosis is primarily governed by three essential components: reactive oxygen species (ROS), the interaction between glutathione (GSH), ROS facilitated by Glutathione Peroxidase 4 (GPX4), and the induction of lipid peroxidation catalyzed by arachidonic acid lipoxygenase (ALOX) (Sui et al., 2022). In the process of ferroptosis, iron triggers an excessive generation of ROS through the Fenton reaction. Therefore, ferroptosis is an iron-dependent process (Rizzollo et al., 2021). Given the extensive array of mechanisms associated with ferroptosis, this summary will focus on the key mechanisms relevant to compounds-induced ferroptosis in BC.

### 2.1 The role of GPX4 and system xc

GPX4 is a selenoenzyme that plays a crucial role in inhibiting ferroptosis by converting toxic phospholipid hydroperoxides (PLOOHs) into non-toxic phospholipid alcohols (PLOHs) (Yang et al., 2014). The system xc-consists of two components: a light chain (xCT, transcribed from SLC7A11) and a heavy chain (4F2, transcribed from SLC3A2) (Huang et al., 2005). This system functions by exporting glutamate out of the cell while simultaneously importing cystine into the cell in a 1:1 ratio (Sato et al., 1999). Once inside the cell, cystine is rapidly converted to L-cysteine, which serves as a crucial building block for the synthesis of intracellular glutathione (GSH). GSH is the most abundant and ubiquitous antioxidant in cells. It plays a vital role in restoring the intracellular reduction-oxidation (REDOX) balance following the generation of ROS. And it is also a necessary substrate for GPX4 enzymatic activity (Niu et al., 2021). Erastin and RSL3 are the classical ferroptosis inducer. Erastin inhibits GPX4 activity by depleting cellular GSH, while RSL3 directly targets and inhibits GPX4 activity (Yang et al., 2014). Cells expressing high levels of SLC7A11 and SLC3A2 are capable of sustaining GPX4 expression through selenocysteine biosynthesis. Meanwhile these cells are more resistant to lipid peroxidation-induced cell death (Lee et al., 2021a). Methyltransferase METTL16 boosts GPX4 expression by means of epigenetic modification of N6-methyladenosine (m6A), thus inhibiting ferroptosis and facilitating the progression of BC (Ye et al., 2023). Different subtypes of triple-negative breast cancer (TNBC) exhibit distinct characteristics regarding their susceptibility to ferroptosis treatment, with the luminal androgen receptor (LAR) subtype being particularly sensitive to ferroptosis induction (Yang et al., 2023a).

### 2.2 Iron metabolism

Iron metabolism plays a critical role in ferroptosis, with free iron serving as the foundation for this process (Dixon et al., 2012). Ferritin is a cytosolic iron storage protein. It is composed of two subunits, ferritin heavy chain 1 (FTH1) and ferritin light chain (FTL) (Chen X. et al., 2020). The vast majority of intracellular Fe^2+^ is stored in ferritin in the form of labile iron pool (LIP). Excessive levels of free iron can trigger the peroxidation of polyunsaturated fatty acids (PUFA), leading to the generation of lipid peroxides. This process disrupts the structure of cell membranes and initiates ferroptosis through the Fenton reaction (Dixon et al., 2012). Transferrin (TF) is a vital iron-binding protein that transports extracellular iron into cells by binding to transferrin receptors on the cell membrane. This process involves the participation of two distinct isoforms of transferrin receptors, namely, transferrin receptor 1 (TFR1) and transferrin receptor 2 (TFR2) (Gomme et al., 2005). IRP1 and IRP2, members of the iron regulatory protein (IRP) family, maintain intracellular iron homeostasis by regulating the expression of genes related to iron metabolism (Muckenthaler et al., 2008; Küh and n, 2015). Ferroportin (FPN) functions as an iron efflux pump responsible for expelling intracellular iron from the cell, and it serves as a suppressor of ferroptosis (Su et al., 2020). Yu H et al. (Yu et al., 2019), observed that transferrin receptor (TFRC) expression was significantly higher in ER-tissues compared to ER + tissues, and knockdown of estrogen receptor (ER) increased TFRC expression in BC cells. Additionally, sulfasalazine (SAS) can induce ferroptosis in BC cells, particularly in cells with low ER expression.

### 2.3 Ferroptosis-related autophagy

Autophagy is an intracellular degradation process that transports cellular components to lysosomes for degradation (Feng et al., 2014). Recent studies have revealed that heightened autophagy and lysosomal activity could contribute to the promotion of ferroptosis by facilitating iron accumulation or lipid peroxidation (Zhou et al., 2020). Specifically, autophagy in ferroptosis includes ferritin autophagy, adipoautophagy, clock autophagy, and chaperone-mediated autophagy (Liu et al., 2020). They promote ferroptosis by degrading antiferroptosis regulators, namely, ferritin, lipid droplets, ARNTL, and GPX4 (Tang and Kroemer, 2020). Ferritin autophagy is a process of degrading ferritin through autophagy. The liberation of free iron results in elevated iron levels, triggering the Fenton reaction and subsequent oxidative stress, ultimately leading to ferroptosis (Dowdle et al., 2014; Hou et al., 2016). For example, E3 ubiquitin ligase promotes the degradation or transport of target proteins by linking ubiquitin molecules to specific sites of target proteins. The E3 ligases NEDD4 and NEDD4L inhibit ferroptosis by promoting the degradation of mitochondrial voltage-dependent anion channels (VDAC) and lactotransferrin, respectively. Nuclear receptor coactivator 4 (NCOA4) is a selective cargo receptor for autophagic turnover of ferritin by lysosomes. The depletion of NCOA4 or autophagy-related (ATG, such as ATG3, ATG5, ATG7, and ATG13) can impede ferritin degradation, resulting in the buildup of iron and lipid peroxidation, ultimately leading to ferroptosis (Mancias et al., 2014).

### 2.4 Lipid metabolism

Numerous recent studies have provided compelling evidence linking dysregulated fatty acid (FA) metabolism to cancer development (Röhrig and Schulze, 2016). Notably, the accumulation of PUFA oxides stands as a hallmark of ferroptosis, which represents a distinct form of cell death (Ye et al., 2020). Intracellular ROS readily oxidize PUFA, leading to the formation of lipid peroxides that strongly promote the initiation of ferroptosis (Doll and Conrad, 2017). Among the key regulators of lipid metabolism, LPCAT3 and ACSL4 assume critical roles (Hishikawa et al., 2008; Küch et al., 2014). They effectively facilitate ferroptosis by incorporating PUFAs into cellular phospholipids, particularly phosphatidylethanolamine. Importantly, the inhibition of ACSL4 and LPCAT3 through gene knockdown have been demonstrated to impede ferroptosis (Lee J-Y. et al., 2021). In a study by Doll S et al. (Doll et al., 2017), it is observed that ACSL4 exhibits preferential expression in a subset of basal-like BC cell lines. Additionally, ACSL4 expression closely correlates with the sensitivity of RSL3. RSL3 is a potent inducer of ferroptosis.

### 2.5 FSP1 and CoQ10

Doll S et al. (Doll et al., 2019), have discovered that ferroptosis inhibitor protein 1 (FSP1), previously recognized as AIFM2, serves as a formidable factor in conferring resistance against ferroptosis. Importantly, their findings reveal that FSP1’s protective influence operates autonomously from the GSH-GPX4 pathway. Coenzyme Q10 (CoQ10), often referred to as ubiquinone, is a lipid-soluble antioxidant with significant roles within the cell. One of its primary functions is to actively engage in cellular energy production, particularly by facilitating electron transport along the cellular respiratory chain within mitochondria (Hargreaves et al., 2020). Within the FSP1-NAD(P)H pathway, CoQ10 plays a pivotal role by directly intercepting lipid peroxyl radicals, thereby diminishing lipid peroxides. In this process, FSP1 acts as a catalyst, facilitating the regeneration of CoQ10 at the cost of NAD(P)H (Bersuker et al., 2019). Yang J et al. (Yang J. et al., 2022), effectively countered ferroptosis resistance mediated by FSP1 in TNBC by encapsulating rosuvastatin within silk fibroin nanoparticles, denoted as Cu-SF(RSV) NPs. This approach resulted in the inhibition of TNBC progression.

## 3 Compounds as ferroptosis regulator in breast cancer

### 3.1 Natural bioactive compounds

**FIGURE 1:**
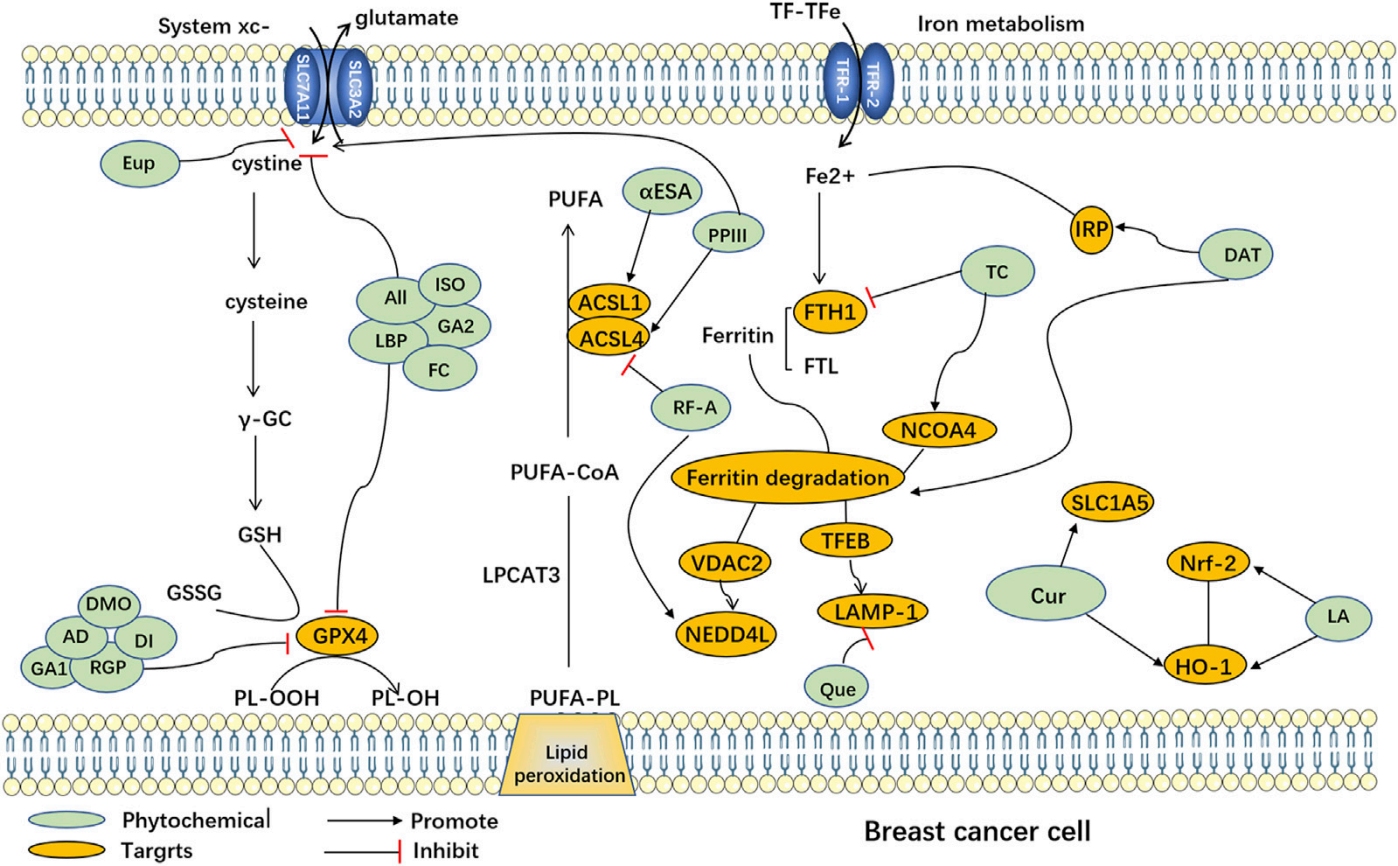
Ferroptosis induced by phytochemicals in breast cancer cells. Abbreviation: AD, Arnicolide D; All, Alloimperatorin; Cur, Curcumin; DAT, Dihydroartemisinin; DMO, DMOCPTL; DT, Dihydroisotanshinone I; Eup, Eupaformosanin; FC, Formosanin C; GA1, Gallic acid; GA2, Glycyrrhetinic acid; ISO, Isoliquiritin; LA, Levistilide A; LBP, Lycium barbarum polysaccharide; PPIII, Polyphyllin III; Que, Quercetin; RF-A, Robustaflavone 7.5′'-dimethyl ether; RGP, Red ginseng polysaccharide; TC, Tetrandrine citrate; αESA, α-eleostearic acid; ACSL1, Acyl-CoA Synthetase Long-Chain Family Member 1; ACSL4, Acyl-CoA Synthetase Long-Chain Family Member 4; FTH1, ferritin heavy chain 1; FTL: ferritin light chain; GSH: Glutathione; GSSG: Glutathione disulfide; HO-1: heme oxygenase-1; IRP: Iron regulatory protein; LAMP-1, Lysosomal-associated membrane protein 1; LPCAT3, Lysophosphatidylcholine acyltransferase 3; NCOA4, nuclear receptor coactivator 4; Nrf-2, nuclear factor erythroid-2-related factor 2; NEDD4L, Neural precursor cell Expressed Developmentally Downregulated 4-Like; PL-OOH, Peroxyl radical lipid hydroperoxide; PL-OH, Phospholipid alcohol; PUFA, Polyunsaturated Fatty Acid; PUFA-CoA, Polyunsaturated Fatty Acid-Coenzyme A; PUFA-PL, Polyunsaturated Fatty Acid-Phospholipid; SLC7A11, Solute Carrier Family 7 Member 11 (xcT); SLC3A2: Solute Carrier Family 3 Member 2; TFEB, Transcription Factor EB; VDAC2, Voltage-Dependent Anion Channel 2; TF-TFe, Transcription Factor Transferrin Enhancer.

Natural plants are a rich source of bioactive components and extracts that exhibit diverse biological activities, including anti-cancer properties (Haque et al., 2021), antioxidative effects, immunomodulation (Zhou X. et al., 2022), and antibacterial actions (Tian et al., 2022). Consequently, they hold tremendous potential for the treatment of various diseases. However, the precise mechanisms and targets involved in ferroptosis remain poorly understood. Recent research has highlighted the potential of numerous natural plant compounds as modulators of ferroptosis, offering a promising avenue for their anti-cancer effects (Zhang S. et al., 2021; Zhao et al., 2022). Phytochemicals can be categorized into different groups based on their chemical composition, including polyphenols, terpenoids, alkaloids, flavonoids, and others (Akhtar et al., 2020). (The natural compounds are summarized in Table 1, and the mechanism of action of them are summarized in Figure 1.)

**TABLE 1:** Phytochemicals that exert anti-breast cancer effects by targeting ferroptosis.

Serial number	Phytochemical compounds	Class of compounds	Source	Effects and mechanisms	Cell/Animal models	References
1	Curcumin	Polyphenols (phenolic acid)	Turmeric	↑HO-1	MCF-7, MDA-MB-231	Li et al. (2020)
				↑SLC1A5	MDA-MB-453, MCF-7; BALB/c nude mice	Cao et al. (2022)
2	Robustaflavone 7.5′'-dimethyl ether	Polyphenols (Flavonoids)	S. trichoclada	↓ACSL4	MCF-7	Xie et al. (2022b)
				↑VDAC2, ↓Nedd4	MCF-7	Xie et al. (2021)
3	Quercetin	Polyphenols (Flavonoids)	Fruits and vegetables, such as onions, grapes and leafy greens	↑TFEB, ↓lysosom, l degradation of ferritin	MCF-7, MDA-231	An and Hu (2022)
4	Levistilide A	Polyphenols (Flavonoids)	Chuanxiong rhizoma	↓NRF-2/HO-1 Signaling Pathway	MCF-7, MDA-MB-231	Jing et al. (2022)
5	Gallic acid	Polysaccharides	A variety of plants, such as gallnuts, watercress	↓GPX4	MDA-MB-231	Khorsandi et al. (2020)
6	Glycyrrhetinic acid	Terpenoids (Triterpenoids)	Licorice	↑NAPDH oxidase activity, ↑iNOS expression, ↓glutathione level, ↓GPX4, ↓SLC7A11	MDA-MB-231, MCF-10A, BT-549	Wen et al. (2021a)
7	Isoliquiritin	Terpenoids (Triterpenoids)	Liquorice	↓GPX4, ↓SLC7A11	MDA-MB-231, MCF-7, BALB/c nude mice	Wang et al. (2023)
8	Eupaformosanin	Terpenoids (Sesquiterpenoids)	Eupatorium cannabinum linn	↓SLC7A11	MDA-MB-231, MDA-MB-468; BALB/c nude mice	Wei et al. (2022)
9	Arnicolide D	Terpenoids (Sesquiterpenoids)	Centipeda minima	↓GPX4	MDA-MB-231, MDA-MB-468	Wen et al. (2021b)
10	Dihydroisotanshinone I	Terpenoids (Diterpenoids)	Salvia miltiorrhiza Bunge	↓GPX4	MCF-7, MDA-MB-231, BALB/c nude mice	Lin et al. (2019)
11	Dihydroartemisinin	Terpenoids (Monoterpenoids)	Artemisia annua	IRP-IRE signaling, ↑lysosomal degradation of ferritin	MDA-MB-453, MCF7	Chen et al. (2020b)
12	DMOCPTL	Terpenoids (Sesquiterpenoids)	Tanacetum parthenium	↓GPX4 (induce ubiquitination)	MDA-MB-231, SUM159, BT574, 4T1, Hs578T, MDA-MB-468	Ding et al. (2021)
13	Lycium barbarum polysaccharide	Polysaccharides	Barbarum	↓ SLC7A11, ↓GPX4	MCF-7, MDA-MB-231	Du et al. (2022)
14	Red ginseng polysaccharide	Polysaccharides	Red ginseng	↓GPX4	MDA-MB-231	Zhai et al. (2022)
15	α-eleostearic acid	Fatty acid compounds (Linolenic acid)	Vernicia fordii	Triggering ACSL1-dependent increase in lipid hydroperoxides	MDA-MB-468, MDA-MB-231, BT-549, BT-20, Hs-578T; NSG mice	Beatty et al. (2021)
16	Tetrandrine citrate	Pyrrole alkaloids	Radix Stephaniae Tetrandrae	↑NCOA4-mediated ferritin phagocytosis, ↓FTH1, ↓GPX4	MDA-MB-231, MCF7	Yin et al. (2022)
17	Alloimperatorin	Benzopyran (Coumarins)	Angelica dahurica	↓GPX4, ↓SLC7A11	MCF-10A, MDA-MB-231, MCF-7	Zhang et al. (2022b)
18	Polyphyllin III	Glycosides (Saponins)	Chinese Clematis	↓KLF4, ↑ACSL4, ↑SLC7A11	MDA-MB-231, HS578T, MCF-7, T47D, HBL-100, BT549, MDA-MB-453, NHFB	Zhou et al. (2021)
19	Formosanin C	Glycosides (Saponins)	Rhizoma Paridis	↓GPX4, ↓SLC7A11, ↑LC3, ↑ferritinophagy	MCF-7, MDA-MB-231	Chen et al. (2022b)

**↑, promote; ↓, inhibit;** ACSL1, Acyl-CoA Synthetase Long-Chain Family Member 1; ACSL4, Acyl-CoA Synthetase Long-Chain Family Member 4; FPN, ferroportin; FTH1, ferritin heavy chain 1; FTL, ferritin light chain; GSH, glutathione; GSSG, glutathione disulfide; HO-1, heme oxygenase-1; IRP, iron regulatory protein; LAMP-1, Lysosomal-associated membrane protein 1; LPCAT3, Lysophosphatidylcholine acyltransferase 3; KLF4, Kruppel-like factor 4; NADPH, nicotinamide adenine dinucleotide phosphate, reduced form; NCOA4, nuclear receptor coactivator 4; NOS, nitric oxide synthase; NRF-2, nuclear factor erythroid-2-related factor 2; NEDD4L, Neural precursor cell Expressed Developmentally Downregulated 4-Like; PL-OOH, peroxyl radical lipid hydroperoxide; PL-OH, phospholipid alcohol; PUFA, polyunsaturated fatty acid; PUFA-CoA, Polyunsaturated Fatty Acid-Coenzyme A; PUFA-PL, Polyunsaturated Fatty Acid-Phospholipid; SLC7A11, Solute Carrier Family 7 Member 11 (xcT); SLC3A2, Solute Carrier Family 3 Member 2; TFEB, Transcription Factor EB; VDAC2, Voltage-Dependent Anion Channel 2; TF-TFe, Transcription Factor Transferrin Enhancer.

#### 3.1.1 Polyphenols

Polyphenols encompass a diverse group of organic compounds characterized by multiple phenolic groups (-OH) arranged in benzene rings or analogous structures. This category includes various bioactive plant metabolites like phenolic acids, flavonoids, lignans, anthocyanins, and others (Williamson, 2017; Cory et al., 2018).

Curcumin is the principal active compound derived from the underground rhizome of the traditional Chinese medicine Curcuma longa. It belongs to the phenolic acid subgroup within the polyphenol family. Studies by Cao, X et al. (Cao et al., 2022), have demonstrated that Curcumin induces ferroptosis in breast cancer cells by upregulating the expression of SLC1A5. The inhibition of SLC1A5 in MDA-MB-453 and MCF-7 cells resulted in decreased glutamine uptake, increased levels of lipid ROS, accumulation of lipid peroxidation products, and elevated intracellular Fe^2+^ levels. Similarly, Li, R et al. (Li et al., 2020), discovered that curcumin upregulates several ferroptosis-related target genes involved in REDOX regulation, particularly HO-1. The effective attenuation of curcumin-induced ferroptosis in breast cancer cells is achieved by inhibiting HO-1 using the specific inhibitor Zinc Protoporphyrin (ZnPP). This is demonstrated by a notable decrease in lipid peroxidation and an elevation in intracellular glutathione levels.

Xie Y et al. (Xie et al., 2022b), isolated various diflavone compounds with distinct structural forms from Seladella. Among them, Robustaflavone 7,5″-dimethyl ether (RF-A) exhibite the ability to induce ferroptosis in breast cancer cells by downregulating ACSL4 protein expression. Furthermore, RF-A promoted ferroptosis by upregulating VDAC2 protein expression and suppressing the expression of NEDD4 E3 ubiquitin ligase, leading to lipid peroxidation and ROS production (Xie et al., 2021).

Quercetin, another polyphenolic compound found in several plants and fruits such as onions, grapes, apples, and leafy green vegetables. It can activate the transcription of transcription factor EB (TFEB) in BC cells. This, in turn, stimulates the lysosomal degradation of ferritin, resulting in increased intracellular iron ion concentration and the induction of ferroptosis. (An and Hu, 2022).

Levistilide A (LA) is an active compound derived from Ligusticum wallichii, which has been shown to significantly enhance ROS-induced ferroptosis by activating the Nrf2/HO-1 signaling pathway (Jing et al., 2022).

Gallic acid (GA1) is a naturally occurring phenolic acid compound widely found in nature (Al Zahrani et al., 2020). Extensive research has revealed the remarkable antioxidant and anticancer properties of GA1. Alkyl gallate, a derivative of GA1, is commonly employed as an antioxidant additive in various food products (Dodo et al., 2008). A study has shown that the combination of low-level laser irradiation and GA1 treatment has been shown to induce ferroptosis in MDA-MB-231 cells by reducing GPX4 activity and promoting lipid peroxidation (Khorsandi et al., 2020).

#### 3.1.2 Terpenoids

Terpenes are natural compounds composed of multiple isoprene units (C5H8). Terpenoids can be classified into different groups based on the number of ring structures they contain. Terpenoids can be categorized into different groups based on the number of ring structures they possess. Monomeric terpenoids consist of a single ring structure, while diterpenoids and triterpenoids are characterized by two and three ring structures respectively (Yang and Dou, 2010). Each of these classes has distinct structures and biological activities.

Glycyrrhetinic acid (GA2), also known as 18-β-glycyrrhetinic acid, is the primary active compound derived from licorice. It exhibits a wide range of anticancer activities in various types of cancer. The cytotoxic and antitumor properties of GA2 are attributed to the presence of triterpene functional groups in its structure, namely, carboxylic acid (-COOH) and hydroxyl (-OH) groups (Roohbakhsh et al., 2016; Hussain et al., 2021). GA2 has been shown to induce the production of reactive oxygen species (superoxide and hydroxyl radicals) by activating NADPH oxidase and iNOS. Additionally, it reduces the antioxidant capacity of cells by down-regulating SLC7A11 expression and inhibiting GPX4. These actions subsequently promote lipid peroxidation mediated by reactive oxygen/nitrogen species (ROS/RNS) and trigger ferroptosis (Wen Y. et al., 2021).

Isoliquiritin (ISO) is a triterpenoid compound derived from licorice. Administration of ISO results in elevated levels of intracellular Fe^2+^, ROS, and malondialdehyde (MDA). Concurrently, it reduces the levels of GSH and the relative protein expression of GPX4 and xCT. It is proposed that ISO regulates ferroptosis by potentially inhibiting NF-κB signaling (Wang et al., 2023).

Eupaformosin is a sesquiterpene lactone compound isolated from Eupatorium cannabinum. *In vitro* and *in vivo* studies have demonstrated that eupaformosin induces ferroptosis by promoting the ubiquitination of mutant p53. p53 is a protein involved in cell cycle regulation and tumor suppression (Wei et al., 2022).

Arnicolide D is a sesquiterpenoid compound extracted from Centipeda minima, a traditional Chinese medicinal plant. Dihydroisotanshinone I is extracted from the dried root of Salvia miltiorrhiza. Both of them can activate ferroptosis by increasing the accumulation of Fe^2+^ and MDA while suppressing the expression of GPX4^[71 72]^.

Dihydroartemisinin (DAT), an active metabolite derived from artemisinin and its derivatives (ARTs), has gained significant recognition as an effective drug for the treatment of malaria in clinical practice. DAT demonstrates enhanced bioavailability when compared to its natural counterpart. However, DAT has recently garnered increasing attention for its potential anticancer activity in addition to its antimalarial properties. Researchers have been intrigued by the promising anticancer effects exhibited by DAT, leading to a growing interest in exploring its therapeutic applications in the field of oncology (Dai et al., 2021). DAT has the ability to induce the degradation of ferritin through lysosomal pathways, consequently impacting iron homeostasis regulated by the iron regulatory protein/iron-responsive element (IRP/IRE) system. This process leads to an increase in the levels of free iron within cells, rendering them more susceptible to ferroptosis (Chen G-Q. et al., 2020).

DMOCPTL is a derivative of the natural product parthenolide found in plants like feverfew. It induces ferroptosis by ubiquitinating GPX4 (Ding et al., 2021). This process leads to the degradation of GPX4 and subsequent disruption of cellular antioxidant defense mechanisms.

#### 3.1.3 Other classes of natural compounds

There are other various classes of compounds that exhibit different effects on ferroptosis. These include two polysaccharides, one alkaloid, one Benzopyran (Coumarins), one Fatty acid compound (linolenic acid), and one Glycoside (Saponins).

Lycium barbarum polysaccharide (LBP) is extracted from the fruit of Chinese medicine Lycium barbarum. LBP induced ferroptosis by reducing the expression levels of SLC7A11 and GPX4 (Du et al., 2022).

Red ginseng polysaccharide (RGP) is one of the main active ingredients extracted from red ginseng. Similar to Lycium barbarum polysaccharide, red ginseng polysaccharide induced ferroptosis by reducing GPX4 expression. And the overexpression of GPX4 in TNBC cells would make cancer cells resistant to RGP (Zhai et al., 2022).

Alpha eleostearic acid (αESA) is extracted from tung seed of conjugated linoleic acid ester (Cui et al., 2018). αESA was found to induce ferroptosis *in vitro* by triggering an ACSL1-dependent increase in lipid hydroperoxides. At the same time, they administered tung oil to BC mice by gavage and found that tung oil significantly inhibited tumor growth. Gene sequencing of mice revealed a significant upregulation of ferroptosis-related genes, indicating that the inhibition of tumor growth *in vivo* was associated with cell death caused by αESA *in vitro* (Beatty et al., 2021).

Tetrandrine citrate (TetC) is a new tetrandrine salt with high water solubility, which can be extracted from the traditional Chinese medicine Tetrandrine. It drove BC cell ferroptosis by activating NCOA4-mediated ferritin phagocytosis and inhibiting GPX4 expression in BC cells (Yin et al., 2022).

Alloimperatorin is a coumarin compound extracted from Angelica dahurica, a traditional Chinese medicine. It had been found to promote the accumulation of Fe^2+^, ROS, and malondialdehyde. Meanwhile, it significantly reduced the mRNA and protein expression levels of SLC7A11 and GPX4. These findings indicate that alloimperatorin induces ferroptosis (Zhang J. et al., 2022).

Polyphyllin III is a natural saponin extracted from Chinese medicine Paris flos. Zhou Y et al. (Zhou et al., 2021), found that it exerted a proliferation inhibitory effect on MDA-MB-231 triple-negative BC cells mainly through ACSL4-mediated elevation of lipid peroxidation and induction of ferroptosis. At the same time, the study also found that Polyphyllin III treatment induced a protective upregulation of xcT, a negative regulator of ferroptosis.

Formosanin C (FC) is a diosgenin glycoside extracted from Rhizoma paridis with anticancer activity (Man et al., 2011). FC induces both autophagy and apoptosis in human lung cancer cells and multiple myeloma cells (Chen P. et al., 2022; Chu et al., 2023). Meanwhile, Chen HC et al. (Chen HC. et al., 2022), identified that FC was a potent ferroptosis inducer in MDA-MB-231 Cells. FC-induced ferroptosis in TNBC is associated with suppressed levels of the FPN, xcT, and GPX4. Ferritinophagy also plays an important role in FC-induced ferroptosis. In addition, FC enhanced the sensitivity of TNBC cells to cisplatin.

### 3.2 Chemically synthesized compounds

**FIGURE 2:**
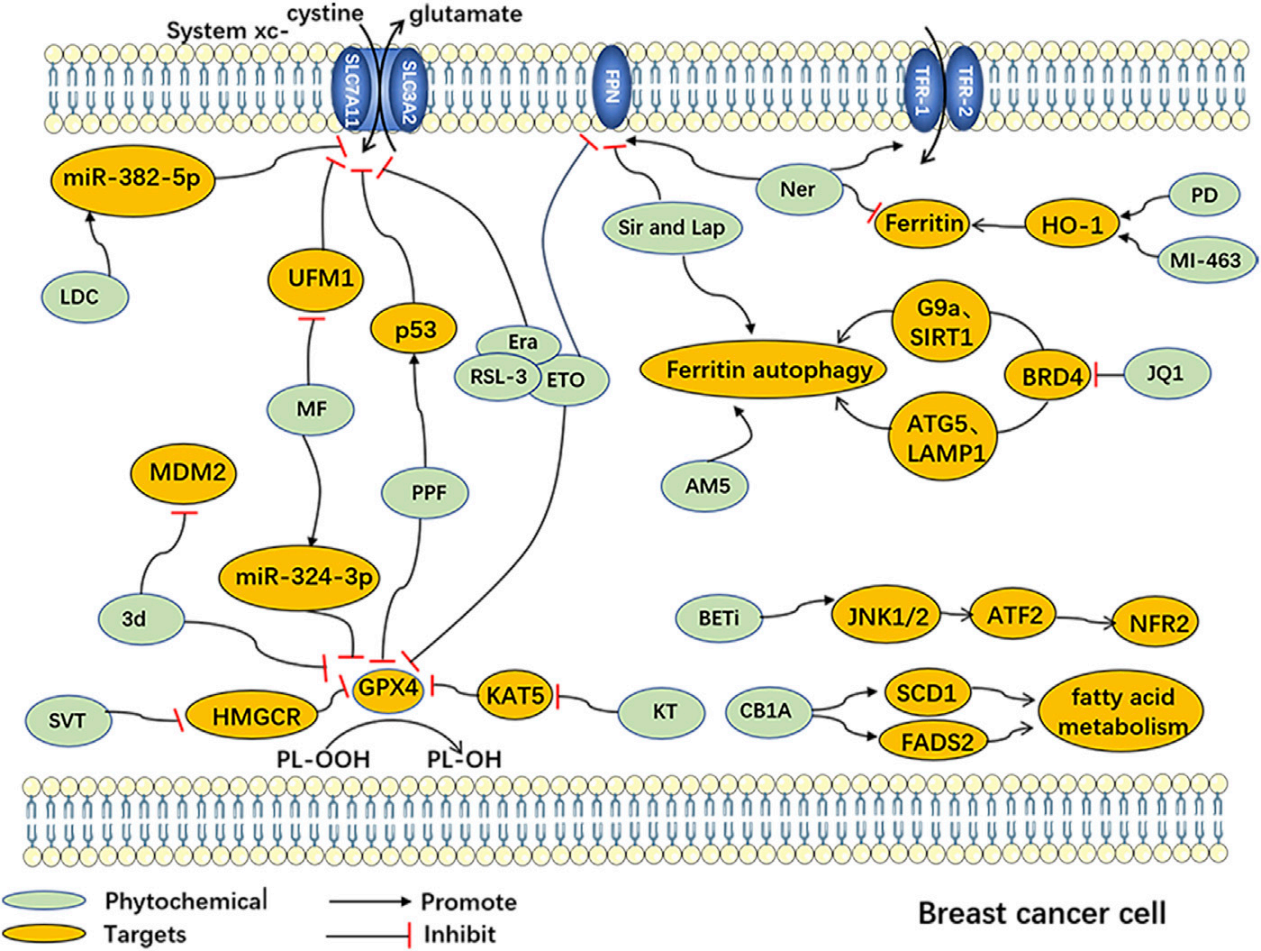
Ferroptosisinduced by Chemically synthesized drugs in breast cancer cells Abbreviation: AM5, Ironomycin; 3d, 3'-(nitroisoxazole)spiro [pyrrolidin-3.2′-oxindoles]; CB1A: CB1 antagonists; Era, Erastin; ETO, Etoposide; BETi, BET inhibitor; LDC, Lidocaine; JQ1, (+)-JQ1, bromodomain and extra-terminal motif inhibitor; KT, Ketamine; MF, Metformin; MI-463, Menin-MLL inhibitors; Ner, Neratinib; PD, Phenazine derivatives; PPF, Propofol; RSL-3, Ras-selective lethal 3; Sir and Lap, Lapatinib and Siramesine; SVT, Simvastatin; ATF-2, Activating Transcription Factor 2; ATG-5, Autophagy-related gene 5; BRD4, Bromodomain-containing protein 4; FADS2, Fatty Acid Desaturase 2; FPN, ferroportin; G9a, Euchromatic Histone Lysine Methyltransferase 2; HMGCR, 3-hydroxy-3-methylglutaryl-CoA reductase; HO-1, heme oxygenase-1; JNK1/2, c-Jun N-terminal kinase 1/2; KAT5, Lysine acetyltransferase 5; LAMP1, Lysosomal-associated membrane protein 1; MDM2, Mouse Double Minute 2; miR-324-3p, miRNA-324-3p; miR-382-5p, miRNA-382-5p; NFR-2, Nuclear factor erythroid 2-related factor 2; SCD1, Stearoyl-CoA desaturase 1; SIRT1, Sirtuin 1; UFM1, Ubiquitin-fold modifier 1.

Additionally, several chemosynthetic compounds have demonstrated anti-breast cancer effects through the targeted induction of ferroptosis in BC cells. Notable examples of ferroptosis inducers are erastin and RSL-3, which are widely recognized in the field. Among these are drugs that have already received clinical approval and have been extensively utilized in other fields. Examples include metformin and simvastatin. Furthermore, new chemically synthesized compounds, such as BET inhibitors (BETi), have also shown promising potential in this regard. (The chemically synthesized compounds. are summarized in Table 2, and the mechanism of action of them are summarized in Figure 2.)

**TABLE 2:** Chemically synthesized drugs that act against breast cancer by targeting ferroptosis.

Serial number	Name of drug	Drug combination	Effects and mechanisms	Cell/Animal models	References
1	Erastin	——	↓ SLC7A11, ↓GPX4	MDA-MB-231, CAL-120	Lee et al. (2021c)
		TGF-β1	↓the formation of the BECN1-SLC7A11 complex	MDA-MB-231, MCF-7	Huang et al. (2023)
2	RSL3	——	↓ SLC7A11, ↓GPX4	MDA-MB231, CAL-120	Lee et al. (2021c)
3	Simvastatin	——	↓HMGCR, ↓MVA pathway, ↓GPX4	MDA-MB-231, MCF-7	Yao et al. (2021b)
		——	↓GPX4	MCF-7, MDA-MB-231	Elakkad et al. (2021)
4	Metformin	SAS	↓SLC7A11, ↓UFM1, ↓UFMylation	MCF-7, T47D, HCC1937, Bcap37, NHFB, HBL-100/nude mice	Yang et al. (2021)
		——	↑miR-324-3p, ↓GPX4	MCF-7, MDA-MB-231	Yan and Bu (2021)
			↓H19, ↑autophagy	MCF-7	Chen et al. (2022c)
5	Lapatinib and Siramesine	——	↑transferrin, ↓FPN, ↓SLC7A11, ↓GPX4	MCF-7, MDA-MB-231, SKBr3	Ma et al. (2016)
6	Neratinib	——	↓Ferritin, ↑Ferroportin-1	TBCP-1, SKBR3, 67NR, 4T1.2/BALB/C nude mice	Nagpal et al. (2019)
7	Etoposide	erastin	↓SLC7A11, ↓GPX4, ↑IREB2, ↓FPN1, ↑ACSF2	MCF-7, MCF-10A, MDA-MB-231	Ozkan and Bakar-Ates (2023a)
8	Ketamine	——	↓KAT5, ↓GPX4	MCF-7, T47D	Li et al. (2021)
9	Propofol	Doxorubicin/Taxol	↑p53, ↓GPX4, ↓SLC7A11	MDA-MB-231	Sun et al. (2022)
10	Lidocaine	——	↑miR-382-5p, ↓SLC7A11	T47D	Sun et al. (2021a)
11	Ironomycin (AM5)	——	↑the degradation of ferritin in lysosomes	MCF-7, HMLER	Mai et al. (2017)
12	(+)-JQ1	erastin/RSL3/Sorafenib	↓FTH1, ↑ATG5, ↑LAMP1, ↑ferritinophagy, ↓G9a, ↑SIRT1, ↑DNA methylation	MDA-MB-231, Hs578T, A549, H1299	Sui et al. (2019)
13	BET inhibitor	BTZ	↑JNK1/2, ↑ATF2, ↑NRF2	MDA-MB-231, BT-549, HeLa, A549/nude mice	Wang et al. (2021)
14	Phenazine derivatives	——	↑IRP2, ↑TFR1, ↓ferritin	Breast cancer stem cells	Yang et al. (2022b)
15	CB1 antagonists	erastin/RSL3	↑SCD1, ↑FADS2	MDA-MB-231, HCC1937	Li et al. (2022)
16	MI-463	auranofin	↑HO-1	BT-549, MDA-MB-468, MDA-MB-231, MCF-7, T47D	Kato et al. (2020)
17	3'-(nitroisoxazole) spiro [pyrrolidin-3.2′-oxindoles]	——	↓MDM2, ↓GPX4	MCF-7	Liu et al. (2021)

**↑, promote; ↓, inhibit;** ACSF2, Acyl-CoA synthetase family member 2; ATF-2, Activating Transcription Factor 2; ATG-5, Autophagy-related gene 5; BRD4, Bromodomain-containing protein 4; FADS2, Fatty Acid Desaturase 2; FPN, ferroportin; FTH1, ferritin heavy chain 1; FTL, ferritin light chain; G9a, Euchromatic Histone Lysine Methyltransferase 2; HMGCR, 3-hydroxy-3-methylglutaryl-CoA, reductase; HO-1, heme oxygenase-1; IREB2, Iron-responsive element-binding protein 2; JNK1/2, c-Jun N-terminal kinase 1/2; KAT5, Lysine acetyltransferase 5; LAMP1, Lysosomal-associated membrane protein 1; MDM2, Mouse Double Minute 2; miR-324-3p, miRNA-324-3p; miR-382-5p, miRNA-382-5p; MVA, mevalonate; NFR-2, Nuclear factor erythroid 2-related factor 2; SCD1, Stearoyl-CoA desaturase 1; SIRT1, Sirtuin 1; TFR1, Transferrin receptor 1; UFM1, Ubiquitin-fold modifier 1.

#### 3.2.1 Erastin and RSL-3

In 2003, Dolma et al. (Dolma et al., 2003), classic apoptotic features were not found in erastin-induced cell death. Erastin-induced cell death is considered as a novel form of non-apoptotic cell death. Dixon et al. (Dixon et al., 2012), named erastin-induced cell death as ferroptosis. At the same time, as GSH is an indispensable substrate for the antioxidant effect of GPX4, the activity of GPX4 will also be inhibited (Zhao et al., 2020). Huang P et al. (Huang et al., 2023), found that the combination treatment of erastin and TGF-β1 could mediate autophagy-dependent ferroptosis by inducing the formation of the BECN1-SLC7A11 complex. Meanwhile, Lee N et al. (Lee et al., 2021c), also found that the classical ferroptosis inducer RSL-3 was able to induce the occurrence of ferroptosis in breast cancer cells.

#### 3.2.2 Statins

In recent studies, there is mounting evidence indicating the potential of statins as agents capable of inducing ferroptosis (Yao X. et al., 2021; Elakkad et al., 2021; Ning et al., 2021; Zhang Q. et al., 2022). Statins are potent inhibitors of HMGCR, which plays a crucial role in controlling cholesterol biosynthesis through the mevalonate pathway (MVA) (Yu et al., 2017). The mevalonate pathway is involved in the synthesis of selenoproteins, including the active center of GPX4. By inhibiting the mevalonate pathway, statins can regulate ferroptosis by simultaneously suppressing ROS and down-regulating the levels of GPX4 (Yao X. et al., 2021; Elakkad et al., 2021). Notably, YAO X et al. (Yao X. et al., 2021), demonstrated an enhanced therapeutic effect against TNBC by utilizing zwitterionic polymer-coated magnetic nanoparticles (Fe3O4@PCBMA) to deliver the ferroptosis-inducing drug simvastatin (SIM).

#### 3.2.3 Metformin

Metformin is the most widely used oral hypoglycemic drug in clinical practice, which is commonly used to treat type 2 diabetes. In recent years, metformin has been proved to have the effect of cancer treatment and prevention, such as liver cancer, BC, colon cancer, pancreatic cancer and so on (Heckman-Stoddard et al., 2017). The verification of clinical efficacy of metformin and the exploration of potential mechanisms have become the research hotspots in the field of cancer. Metformin can activate AMPK to inactivate mTOR, thereby reducing cell proliferation and inducing apoptosis and cell cycle arrest (Zhao et al., 2015; Heckman-Stoddard et al., 2017). At the same time, metformin can indirectly affect tumorigenesis by affecting insulin levels and inducing energy stress (Pernicova and Korbonits, 2014). In addition, recent studies have found that metformin can also exert anti-tumor effects by inducing ferroptosis. Yang J et al. (Yang et al., 2021), found that metformin reduced the protein stability of SLC7A11 by inhibiting its UFMylation process. In addition, metformin could act synergistically when combined with the xc-system inhibitor sulfasalazine. Non-coding RNA are a class of RNA molecules that are widespread in cells and are synthesized during transcription but not translated into proteins. Non-coding RNA can be divided into several subclasses, including long non-coding RNA (lncRNA), microRNA (miRNA), small interfering RNA (siRNA), small nuclear RNA (snRNA) and so on (Yan and Bu, 2021). Metformin was found to induce ferroptosis via non-coding RNA. In TNBC cells, metformin promoted ferroptosis by up-regulating miR-324-3p expression and targeting GPX4 inhibition (Hou et al., 2021). Meanwhile, metformin-induced ferroptosis is achieved through the downregulation of lncRNA H19 and the inhibition of autophagy (Chen J. et al., 2022).

#### 3.2.4 Anti-tumor drugs

Anti-tumor drugs encompass various types, including chemotherapy drugs and targeted drugs. Lapatinib has been approved for the treatment of ErbB-positive BC (Tolaney, 2014). It functions as a dual tyrosine kinase inhibitor, targeting both ErbB1 and ErbB2 tyrosine kinase receptors (Wood et al., 2004). On the other hand, siramesine is a sigma-2 receptor ligand originally developed for depression treatment (Heading C. Siramesine H Lundbeck, 2001). S MA et al. (Ma et al., 2016), discovered that the combination of siramesine and lapatinib induced ferroptosis by increasing the expression of transferrin and decreasing the expression of ferroportin-1 (FPN1). Meanwile, they found that cystine transport inhibition promotes siramesine and lapatinib-induced cell death.

Neratinib, an irreversible pan-TKI, targets EGFR/HER1, HER2, and HER4 of the ERBB tyrosine kinase family (Rabindran et al., 2004). It is approved for extended adjuvant therapy in HER2-positive breast cancer as a single agent and in combination with capecitabine for advanced or metastatic disease (Geyer et al., 2006; Kourie et al., 2017). In HER2-positive BC cells (TBCP-1) treated with Neratinib, the levels of ferritin and FPN1 increased by 2-fold and 1.6-fold, respectively. Intracellular iron concentration also showed a dose-dependent increase at 72 h, as demonstrated by ICP-MS analysis. These findings indicated the proferroptosis activity of neratinib in HER2-positive BC cells (Nagpal et al., 2019).

Etoposide is a semi-synthetic derivative of podophyllotoxin. It is commonly used to treat malignant tumors such as leukemia and lymphoma (Meresse et al., 2004). Its primary mechanism of action involves inhibiting cell growth by forming a complex with topoisomerase II (Baldwin and Osheroff, 2005). A recent study (Ozkan and Bakar-Ates, 2023a) discovered that the combination of etoposide and erastin synergistically induced ferroptosis. This effect may be achieved through the inhibition of glutathione peroxidase activity and alterations in the expression of the ferroptosis regulatory protein IREB2 and FPN1. IREB2 is a protein that responds to changes in intracellular iron levels by binding to IRE, which regulate the expression of ferritin and TFR.

#### 3.2.5 Narcotic drugs

Recent studies have unveiled numerous non-anesthetic effects of anesthetics, including anti-tumor activity, anti-inflammatory properties, and even an antidepressant effect (Caracas et al., 2009; Li et al., 2019; Smith-Apeldoorn et al., 2022). Among them, propofol stands out as one of the most commonly used intravenous anesthetics in clinical practice. Several investigations have indicated that the administration of propofol during surgery improved the prognosis of BC patients compared to inhaled anesthetics (Yap et al., 2019). Sun C et al. (Sun et al., 2022), discovered that propofol, propofol injection emulsion, and phosphopropofol disodium significantly enhanced the antitumor effects of doxorubicin and paclitaxel while inducing changes associated with ferroptosis. In MDA-MB-231 cells, propofol may promote ferroptosis through the p53-SLC7A11-GPX4 pathway.

Ketamine is a rapid-acting anesthetic widely utilized in surgery since 1970, possessing potential anti-tumor activity (Dundee et al., 1970). Its anti-cancer properties have been demonstrated in various cancer models (Duan et al., 2019; He et al., 2021). In a study by Li H et al. (Li et al., 2021), edu-positive breast cancer cells were treated with ketamine, resulting in a significant increase in ferroptosis markers. Ketamine inhibited the expression of GPX4 by attenuating KAT5 in the GPX4 promoter region. This attenuation led to the inhibition of histone H3 lysine 27 acetylation (H3K27ac) and RNA polymerase II (RNA pol II) enrichment, ultimately suppressing the expression of GPX4.

Lidocaine is a local anesthetic extracted from cocaine and widely used in clinical practice. Through the analysis of clinical samples from ovarian and breast cancer patients, Sun P et al. (Sun D. et al., 2021), discovered a downregulation of miR-382-5p expression, while observing an upregulation of SLC7A11 expression. Lidocaine downregulated SLC7A11 expression by enhancing miR-382-5p in the BC cells.

#### 3.2.6 Other novel compounds

Salinomycin is a broad-spectrum antibiotic with antibacterial effects. Mai T et al. (Mai et al., 2017), found a synthetic derivative of salinomycin, which they named ironomycin (AM5). AM5 exhibited a more potent and selective activity against breast CSCs *in vitro* and *in vivo*, accumulates and sequesters iron in lysosomes. In response to the ensuing cytoplasmic depletion of iron, cells triggered the degradation of ferritin in lysosomes, leading to further iron loading in this organelle.

BETi belong to a class of compounds that effectively inhibit the BET protein family. By competing with acetylated histones for binding to BET proteins, BET inhibitors reduce oncogene expression, thereby inhibiting tumor cell proliferation and promoting apoptosis (Andrieu et al., 2016). The BET protein family comprises BRD2, BRD3, BRD4, and BRDT (Shi et al., 2014). Prominent examples of BETi include JQ1 and I-BET. Sui S et al. (Sui et al., 2019), discovered that (+)-JQ1 inhibited BRD4 expression by suppressing the histone methylase G9a or activating the histone deacetylase SIRT1. This inhibition of BRD4 expression enhanced the expression of ATG5 and LAMP1, leading to the induction of ferritin phagocytosis. Wang L et al. (Wang et al., 2021), observed that BEi JQ1 and I-BET 151 (I-BET) activated the JNK/ATF2 pathway in MDA-MB-231 breast cancer cells. Activation of ATF2 by BETi blocks BETi-induced ferroptosis in cancer cells by upregulating NRF2 expression. These findings suggested that inhibiting ATF2 or NRF2 may enhance BET-induced ferroptosis, thereby augmenting the anticancer effects of BETi.

Phenazine derivatives belong to a substantial class of planar nitrogen-containing heterocyclic compounds. The key core structure is the pyrazine ring with two cyclic benzenes (1,4-diazabenzene) (Yan et al., 2021). These compounds exhibit diverse biological activities, including antimicrobial, antidepressant, anticancer, and other pharmacological effects (Gao et al., 2013; Krishnaiah et al., 2018). Yang Y et al. (Yang Y. et al., 2022), conducted a screening of phenazine library compounds targeting breast cancer stem cells and identified three compounds (CPUL119, CPUL129, and CPUL149). These compounds effectively accumulate and sequester iron in lysosomes through their interaction with iron. Additionally, they regulate the expression of proteins involved in iron transport and storage (IRP2, TFR1, ferritin), triggering ferroptosis.

CB1 antagonists block the interaction of endocannabinoids, such as the endogenous CB1 agonist anandamide, with CB1 receptors by binding to the CB1 receptor. Li P et al. (Li et al., 2022), identified that CB1 antagonists regulated sensitivity of TNBC cells to ferroptosis by regulating SCD1-and FADS2-dependent fatty acid metabolism. Some CB1 antagonists, such as rimonabant, have been investigated in clinical trials for obesity, metabolic diseases, and drug abuse. However, rimonabant is no longer approved for medical use due to its psychiatric side effects, such as anxiety and depression (Christensen et al., 2007).

MI-463 is menin-mixed-lineage leukemia inhibitors, unexpectedly inducing the ferroptotic cell death of several cancer cell lines. MI-463 in combination with auranofin, a thioredoxin reductase inhibitor, induced ferroptosis through upregulation of HO-1 (Kato et al., 2020).

Furthermore, there are other compounds, such as nitroisoxazole-containing spiro [pyrrolidin-oxindole] derivatives (Liu et al., 2021). which have been identified as novel dual inhibitors of GPX4 and MDM2.

## 4 Conclusion and perspectives

Ferroptosis, as a form of regulated cell death, offers a promising approach for cancer treatment with multiple regulatory pathways. This review focuses on natural phytochemicals and chemosynthetic compounds that target ferroptosis in BC, summarizing their mechanisms of action both *in vitro* and *in vivo*. Among the various targets, GPX4 and xc-T have emerged as common targets for compound-induced ferroptosis. Some compounds induce ferroptosis also by affecting ferritin autophagy and lipid peroxidation. Transcription factors (P53, NRF2) and non-coding RNA are identified as potential key regulators of compound-induced ferroptosis.

In addition, this review highlights the potential synergistic effects of combining compounds with classical ferroptosis inducers (erastin/RSL3/SAS) or antitumor drugs (Sorafenib/Doxorubicin/paclitaxel) ^[99,113 118,123 127,133]^. For example, Ozkan, E. et al. (Ozkan and Bakar-Ates, 2023b), investigated the combination therapy of etoposide and erastin, demonstrating that ferroptosis induction enhances the anticancer effect of etoposide at lower doses, thereby minimizing side effects on normal tissues. The relationship between ferroptosis and tumor immunity has also gained recognition, with evidence suggesting its involvement in T-cell-mediated antitumor immunity and its impact on the efficacy of cancer immunotherapy (Wang et al., 2019). Recent studies have shown that combining GPX4 inhibitors with immune checkpoint blockade (anti-PD-1) exhibits superior effectiveness in inhibiting breast tumor growth compared to monotherapy (Yang et al., 2023b). Notably, the induction of ferroptosis, either directly or indirectly, holds promise as a combinatorial strategy to enhance anti-PD-1/PD-L1 immunotherapy (Sun LL. et al., 2021). The combination of different drugs to achieve improved efficacy and reduced toxicity represents a potential direction for the development of ferroptosis-targeting drugs in the future.

Despite the rapid progress in understanding the mechanisms of ferroptosis in BC, its clinical application still faces challenges. Ferroptosis occurs not only in cancer cells but also in normal tissues, raising concerns about potential complications upon the use of ferroptosis-inducing agents in humans (Sui et al., 2022). Erastin, a classical ferroptosis inducer, has been shown to alter blood index values and cause mild cerebral infarction and increased glomerular volume in animal experiments (Zhao et al., 2021). Additionally, erastin’s poor water solubility and unstable metabolism *in vivo* make it unsuitable for direct *in vivo* use (Zhao et al., 2020). Although some approved drugs such as metformin and propofol have been shown to induce ferroptosis, their primary therapeutic roles lie outside of ferroptosis induction, and the activation of other pathways by these drugs may lead to significant side effects. It's important to note that current ferroptosis research primarily relies on cell and animal experiments, which may differ from human physiological conditions (Zhang S. et al., 2021). At present, there is no drug study on ferroptosis in BC in the clinical trial registration website. Therefore, caution should be exercised when extrapolating findings from cell and animal studies to human applications. In recent times, nanoparticles have found increasing utility in cancer therapy, demonstrating significant promise as a therapeutic approach. Nanomaterials possess favorable tumor-targeting characteristics that can be effectively combined with ferroptosis drugs. This synergy aims to minimize drug toxicity while augmenting therapeutic efficacy in the treatment of cancer (Xiong et al., 2019; Chen et al., 2023).

Natural phytochemicals found in herbs, cereals, seeds, and other plants offer a potential alternative, as they possess bioactive compounds with protective or disease-preventing properties (Bak et al., 2016). Compared to synthetic compounds, natural phytochemicals are known for their lower toxicity, fewer side effects, higher safety, and diverse chemical profiles (Kapinova et al., 2018). Exploring the vast array of natural phytochemicals holds great potential for discovering effective ferroptosis inducers.

## Figures and Tables

**Table:** 

**ACSL**	Acyl-CoA synthetase long-chain family member
**ALOX**	arachidonic acid lipoxygenase
**ARNTL**	aryl hydrocarbon receptor nuclear translocator-like protein
**ATG**	autophagy-related
**>AIFM2**	apoptosis-inducing factor mitochondrial 2
**AM5**	ironomycin
**AMPK**	AMP-activated protein kinase
**BC**	Breast cancer
**BET**	bromodomain and extra-terminal domain
**BETi**	BET: bromodomain and extra-terminal domain inhibitors
**BECN1**	Beclin 1
**BRD**	bromodomain
**CoQ10**	Coenzyme Q10
**C5H8**	composed of multiple isoprene units
**DAT**	Dihydroartemisinin
**ERBB**	erythroblastic oncogene B
**ER**	estrogen receptor
**FTH1**	ferritin heavy chain 1
**FTL**	ferritin light chain
**FPN**	Ferroportin
**FA**	fatty acid
**FSP1**	ferroptosis inhibitor protein 1
**FC**	Formosanin C
**Fe3O4@PCBMA**	polymer-coated magnetic nanoparticles
**GPX4**	glutathione peroxidase 4
**GSH**	glutathione
**GA**	Gallic acid
**HO-1**	heme oxygenase-1
**HMGCR**	3-hydroxy-3-methylglutaryl coenzyme a reductase
**HER**	human epidermal growth factor receptor
**H3K27ac**	H3 lysine 27 acetylation
**KAT5**	kacetyltransferase 5
**IRP**	members of the iron regulatory protein
**ISO**	Isoliquiritin
**lncRNA**	long non-coding RNA
**IREB**	iron response element-binding protein
**IRE**	iron-responsive element
**lncRNA**	long non-coding RNA
**LIP**	labile iron pool
**LPCAT3**	lysophosphatidylcholine acyltransferase 3
**LA**	Levistilide A
**LAR**	Luminal androgen receptor
**LBP**	Lycium barbarum polysaccharide
**m6A**	N6-methyladenosine
**mTOR**	mammalian target of rapamycin
**miRNA**	microRNA
**MDA**	malondialdehyde
**MVA**	mevalonate
**MDM2**	mouse double minute 2
**NCOA4**	Nuclear receptor coactivator 4
**NEDD4**	neural precursor cell expressed developmentally downregulated 4
**NEDD4L**	neural precursor cell expressed developmentally downregulated 4-like
**PLOOHs**	phospholipid hydroperoxides
**PLOHs**	phospholipid alcohols
**PUFAs**	polyunsaturated fatty acids
**RGP**	Red ginseng polysaccharide
**RNA pol II**	RNA polymerase II
**ROS**	reactive oxygen species
**REDOX**	reduction-oxidation
**RSL3**	ras selective lethal 3
**RF-A**	Robustaflavone 7.5″-dimethyl ether
**siRNA**	small interfering RNA
**snRNA**	small nuclear RNA
**SIM**	simvastatin
**SLC1A5**	solute carrier family 1 member 5
**SLC7A11**	solute carrier family 7 member 11
**SLC3A2**	solute carrier family 3 member 2
**SAS**	sulfasalazine
**TNBC**	triple-negative breast cancer
**TFR/TFRC**	transferrin receptor
**TetC**	Tetrandrine citrate
**VDAC**	voltage-dependent anion channels
**α-ESA**	Alpha eleostearic acid
**ZnPP**	Zinc Protoporphyrin
